# Principles of Comparative Physiology

**Published:** 1854-10

**Authors:** 


*Principles of Comparative Physiology.* By WILLIAM B. CARPENTER, M.D., F.R.S., Examiner in Physiology and Comparative Anatomy in the University of London ; Professor of Medical Jurisprudence in University College, etc., etc. With 309 wood engravings. A new American from the fourth and revised London edition. Philadelphia : Blanchard and Lea.

For several years past a valuable work has been before the public from the pen of Dr. Carpenter, entitled "Principles of General and Comparative Physiology." The volume before us is a new edition of that part of the former work, relating to *Comparative Physiology*. It is perhaps better entitled to the appellation of a new and greatly enlarged work on that part of the subject. It is a large octavo volume of 752 pages, reprinted in the best style of Blanchard and Lea, of Philadelphia.

It is one of those works which the genuine student will ever peruse with pleasure. Dr. Carpenter is one of the few prolific writers, who combines a fair share of reflection and originality of thought with the qualities of a good compiler. He is full in description, and copious in the detail of facts, without being tedious; and in the present work he has given us a full and well arranged exposition of the actual condition of the science of comparative physiology. This volume is especially valuable to the majority of American students, for it contains a vast amount of valuable matter gathered from the works of other authors, to which they have no access. The work is divided into fifteen chapters, embracing the following topics, viz : "On the General Plan of Organic Structure and Development ;" "General View of the Vital Operations of Living Beings, and their Mutual Relations ;" "Of Aliment, its Ingestion and Preparation ;" "Of Absorption and Imbibition ;" "On the Circulation of the Nutritive Fluid ;" 'On Respiration ;" "On the Exhalation of Aqueous Vapor ;" "On Nutrition ;" "On Secretion and Excretion ;" "Evolution of Light, Heat, and Electricity ;" "On Generation and Development ;" "Of the Sensible Motions of Living Beings ;" "Of the Functions of the Nervous System ;" "Of Sensation, and the Organs of the Senses ;" "Of the Production of Sounds by Animals."

The reader will readily perceive, by this list of subjects, that the work of Dr. Carpenter embraces a wide and very interesting field of study. We are sorry, however, to find it, like his great work on human physiology, strongly imbued with the chemico-physiological doctrines of M. Liebig. This is especially the case with the chapters on Aliment and Digestion, and on Animal Heat.

In relation to the first, he says:

"The general facts which appear to have been substantiated, in regard to the mode in which the animal body is sustained by food, may be thus summed up:

"I. The waste of the tissues, of which *albumen* or *gelatine* is the basis, must be supplied by albuminous compounds, whether these be derived from the animal or from the vegetable kingdom. The amount of this 'waste,' and consequently the demand for albuminous aliment, depends essentially upon the degree of vital activity which has been put forth, and especially upon the exercise of the nervo-muscular apparatus; and therefore, *cæteris paribus*, it is greater in cold-blooded animals in proportion to the *elevation* of the external temperature.

"II. The materials of the *adipose* tissue, and the oleaginous particles which seem requisite in the formative operations of the system generally, are derived, in the carniverous races, from the fatty substances which the bodies of their victims may contain; whilst the herbivorous not only find them in the oleaginos state in their food, but have the power of producing them by the conversion of farinaceous and saccharine matters.

"III. The foregoing statements are applicable to all tribes of animals, 'cold-blooded' as well as 'warm-blooded;' we have now to consider the special case of the latter. In the *carniverous* tribes the 'waste' of the tissues is so great, in consequence of the restless activity which is habitual to them, that it appears to furnish a large proportion of the combustible material required for the maintenance of their proper temperature. The remainder is made up by the fat of the animals upon which they feed; and it is to be observed that the amount of this is much greater in the bodies of animals inhabiting the colder regions of the globe, than in the inhabitants of tropical climates. In the *herbivorous* tribes, the case is different. They are, for the most part, much less active; and the 'waste' of their tissues consequently takes place in a less rapid manner, and is far from supplying an adequate amount of combustible material, especially in cold climates. Their heat is in great part sustained by the combustion of the saccharine and oleaginous elements of their food, which are appropriated to this purpose without having ever formed part of the living tissues; and the demand for these will be larger in proportion to the *depression* of the external temperature, a greater generation of caloric being then required to keep up the heat of the body to its proper standard.

"IV. Hence 'cold-blooded' animals can usually sustain the privation of food longer than warm-blooded; and this more especially when they are kept *cool*, so that they are made to *live slowly;* and death, when at last it does ensue, is consequent upon the general deficiency of nutrition. On the other hand, 'warm-blooded' animals, whose temperature is uniformly high, must always *live fast;* and deprivation of food is fatal to them, not only by preventing the due renovation of their tissues, but also by destroying their power of sustaining their heat. The duration of life under these circumstances depends upon the amount of fat previously stored up in the body, and upon the retardation of its expenditure by external *warmth*, or by the inclosure of the body in non-conducting substances; and there is evidence that, if this be duly provided for, and all unnecessary waste by nervo-muscular activity be prevented, the life of a warm-blooded animal may sometimes be prolonged for many weeks without food."

And on the production of heat in animals, he says:

"We have now to inquire what are the conditions of the evolution of Heat in the animal economy. That many of the nutritive processes are subservient to it, can scarcely be doubted; but it seems peculiarly to depend upon those changes in which the function of respiration is concerned—viz: the union of oxygen derived from the atmosphere, with compound of hydrogen and carbon existing in the living system. Wherever the æration of the blood is extensively and actively carried on, there is a proportionate elevation of temperature; and, on the other hand, wherever the respiration is naturally feeble, or the æration of the blood is checked by disease or accidental obstruction, the temperature of the body falls. Thus, in spasmodic asthma, the temperature of the human body during a paroxysm has been found as low as 82°; in the Asiatic Cholera, a thermometer placed iu the mouth has indicated but 77°; and in Cyanosis, (or "blue disease," arising from malformation of the heart impeding perfect arterialization, § 262), the same low temperature has been observed. Again, whenever the temperature of an animal is, by any extraordinary stimulus, quickly raised above that which it was previously maintaining, it is always in connection with increased activity of the respiratory movements, and increased consumption of oxygen. Thus, during the incubation of bees, the insect, by accelerating its respiration, causes the evolution of heat and the consumption of oxygen to take place at least *twenty* times as rapidly as when in a state of repose. Now it has been seen that arterial blood contains a larger proportion of oxygen than exists in venous blood; whilst, on the other hand, the latter contains a larger proportion of carbonic acid than exists in the former (§ 318); and it seems obvious, therefore, that during the passage of blood through the capillaries, a part of its oxygen has been exchanged for carbonic acid. The source of this product is evidently the union of atmospheric oxygen with carbon, furnished by disintegration of the tissues, or (more directly) by the elements of the food (§ 116); and by this union, caloric must be generated, precisely as by the more rapid union of the same materials in the ordinary process of combustion. Further, since these materials yield hydrogen as well as carbon, and since more oxygen almost always disappears from the air which has been respired, than is contained in its carbonic acid (§ 319), it seems probable (although this cannot be demonstrated) that this element also is subjected to the combustive process, with a further generation of caloric; and that of the water which is exhaled from the lungs, a part has been thus produced Further, there can be no doubt that other combustive processes take place in the system, although these may be the chief; for example, of the sulphur and phosphorus which the albuminous components of the food contain, the greater proportion is thrown off by the urinary excretion (§ 389, viz.) in the condition of phosphoric acid, having been combined with oxygen in the system. When all these actions are taken into account—when the amount of heat that should be generated by the production of the quantity of carbonic acid found in the air expired during a given time, is carefully estimated, and the oxygen which has disappeared is considered as having been similarly employed in other combustive processes, especially in the formation of water—it is found that the total so closely corresponds with the amount of heat *actually* generated by the animal during the same time, that it can scarcely be doubted that this process is the main source of calorification."

In these paragraphs we have not only a repetition of Liebig's theories, but also a close imitation of his mode of reasoning.

Aliments are divided into *nitrogenous* and *non-nitrogenous*, or carbonaceous. The first, especially in all the warm-blooded and higher classes of animals, is appropriated to the nourishment of the tissues of the body, while the latter is simply converted into fat, or burned off with the oxygen received by respiration, to support the temperature of the animal. Such is the theory; but what are the facts on which it rests? They are, first, the greater analogy in composition between the albuminous tissues of the animal and the nitrogenous constituents of food; second, that carbon and oxygen unite to produce combustion and the development of caloric out of the body, and hence it is claimed that their union in the living body, however slow and under whatever circumstances it may take place, would be productive of the same ultimate effects, viz.: the production of sensible caloric; and third, that the necessity for the production of caloric in warm blooded animals being greater in cold climates than warm, it is assumed that the inhabitants of high northern latitudes habitually consume a much larger amount of *non-nitrogenous* or carbonaceous matter, than those between the tropics. These alledged facts are all *general* and bear only a remote relation to the conclusions sought to be deduced from them. It may be strictly true that the composition of muscular structure, for instance, bears a closer analogy to that of animal albumen and vegetable gluten, than to starch, sugar or oils; and yet it by no means proves that the first is nourished *exclusively* by the second. Again, it may be true that certain barbarous tribes in high northern latitudes habitually eat large quantities of whale-blubber and other oleaginous matter, but this fact alone does not prove such oleaginous matter is appropriated exclusively to the support of combustion and animal heat. It would be quite as rational to conclude that they eat this because they can get nothing better, and the fact that they are compelled sometimes to live for many days almost exclusively on such oleaginous substances, and at the same time present a good degree of health, and also fnll nutrition of the muscular and albuminous tissues, rather militates against the idea that nature actually preserves any such arbitrary division of the proximate elements of food, appropriating one exclusively to the nourishment of one set of tissues and another to a different purpose. But it is not really true that the inhabitants of the earth eat carbonaceous food in proportion to the coldness of the climate in which they are placed. On the contrary, we find individuals and whole communities living on almost exclusively carbonaceous food in every climate from the equator to the polls.

Thus, the whale blubber and the white bear's meat, so often eaten by the tribes that roam over the frozen regions of the north, is not any more carbonaceous than the oat-meal of Scotland or the *rice* of India, on which millions feed with scarcely a pound of animal food once a year. And yet the men of Southern Asia, who live almost exclusively on rice, present as strong a development of the muscular and nitrogenous tissues as any other inhabitants of the earth. But as we have already said, such facts relating to whole tribes and communities are too remote and general to be of value in determining the specific appropriation of particular proximate elements of food. This must be done by more direct and careful observation. We became satisfied several years since that the whole theory of Liebig in relation to the *combustion* of the carbonaceous elements of food and the almost exclusive appropriation of the nitrogenous elements to the nourishment of the tissues was essentially erroneous. At the meeting of the American Medical Association in Charleston, S. C., May 1851. we read a paper detailing a series of experiments, the results of which were directly adverse to the views of Liebig and Carpenter. That paper was a few months after published in this Journal. Since that time the experimental researches of M. Bernard, M. Dumas, M. Brown-Sequard and many others. have been making constant inroads upon the Geissen Professor's chemico-physiological doctrines. We deem it a serious fault, both in the present work of Dr. Carpenter and the one on Human Physiology, that the doctrines of Liebig are set forth too much as established truths. In this the author has not displayed that critical research and close analysis which the present state of physiological science demands. Aside from this, however, the volume before us is one of great value. We wish it could be thoroughly read by every American student One of the prevailing faults of our profession in this country, arises from a restricted and narrow field for study. Our students limit themselves too much to the mere text-books of the profession. They study man in his healthy and morbid conditions in a manner too isolated—too much cut off from his connection with, and relations to, the rest of animate nature. Their eyes are fixed too exclusively on a single link in the great chain of living beings. Hence the light which is shed on the physiology of man by the pr gressive development of different species of animals is, to them, lost; and they are apt to embrace as truths, that which a wider scope of investigation would show to be error, or only partial truth. Then again, they fail to gain that enlarged view, that elevation of thought, and that sublime enjoyment which is derived from a survey of nature in her length and breadth, and a clear perception of the adaptation of each item to its appropriate end. We repeat our wish that every American Medical Student would procure Dr. Carpenter's work, and read it carefully. D.

